# Is Lesion of Exner’s Area Linked to Progressive Agraphia in Amyotrophic Lateral Sclerosis with Dementia? An Autopsy Case Report

**DOI:** 10.3233/BEN-2010-0276

**Published:** 2010-11-23

**Authors:** Kenji Ishihara, Hiroo Ichikawa, Yoshio Suzuki, Jun’ichi Shiota, Imaharu Nakano, Mitsuru Kawamura

**Affiliations:** ^1^Department of NeurologyShowa University School of MedicineTokyoJapan; ^2^Department of NeurologyUshioda General HospitalKanagawaJapan; ^3^Department of NeurologyJichi Medical SchoolTochigiJapan

**Keywords:** Amyotrophic lateral sclerosis with dementia (ALS-D), fronto-temporal lobar degeneration with TDP-43 proteinopathy (FTLD-TDP), progressive agraphia, Exner’s area

## Abstract

Agraphia, as a neuropsychological symptom of ALS, especially ALS with dementia (ALS-D), has recently attracted more attention. However, the brain lesion responsible has not been identified. Here we present an autopsy case of ALS-D of a patient with obvious agraphia, without aphasia, that also presented cerebrospinal degeneration with TDP-43-pathology compatible with ALS-D. Of the pre-motor frontal lobe cortices, degeneration and immuno-histochemical pathology were most obvious in the caudal area of the left middle frontal gyrus, or Exner’s area. Assuring this area plays a pivotal role in the kanji and kana formation used in writing the Japanese language, this case of ALS-D showed both agraphia and Exner's area stressed pathological lesions. It may thus be the first case to indicate an intimate relationship between the neuropsychological symptoms and an associated lesion for ALS-D.

## 1. Introduction

Amyotrophic lateral sclerosis with dementia (ALS-D) is a nosological condition presenting motor neuron disease (MND) and dementia. The clinical features of dementia in ALS-D are of the frontal lobe type, and ALS-D is located within a framework of fronto-temporal lobar degeneration (FTLD) [1]. Although analysis of language function in ALS-D is considered difficult to carry out, due to severe bulbar palsy, we previously reported that writing disorder may exist in the early stage [2]. Another report, in Japanese, also describes writing disorder in a patient with ALS-D [3]. Here we describe an autopsied case of ALS-D, presenting progressive agraphia without aphasia, and discuss its clinicopathological relationship.

## 2. Case history

A 73 year-old Japanese woman, with a history of breast cancer, was admitted to our hospital with speech disorder and personality change. Her family history provided no clues as to onset of these changes. Six months before admission, she became disoriented about dates and resistant to correction, and after three months, while playing cards with her family, she began missing her turn and became argumentative about continuing. At about the same time, her speech began to be slurred and became difficult, and her activities were slow down.

On admission, she showed no abnormality except for evidence of the surgery for breast cancer. She was cooperative during the neurological examination and

ISSN 0953-4180/10/$27.50 © 2010 – IOS Press and the authors. All rights reserved cognizant of time and place. Her answers to questions were cogent. She could name objects and repeat sentences correctly, although her speech was slurred, slow-pitched, monotonous, and difficult. The tongue showed no atrophy nor fasciculation. Her facial expressions were fairly normal. Although there was no amyotrophy in the extremities, muscle tone was spastic, especially in the lower extremities. Jaw jerk and tendon reflex were exaggerated. Pathological grasping reflex, Hoffman reflex and Trömner reflex were induced bilaterally. Planter reflex was flexor bilaterally. No cerebellar ataxia was observed. As is mentioned later, her writing errors were, however, of great interest. She was not conscious of her illness and scored 7/18 on the frontal assessment battery [4]. Hasegawa's dementia rating scale (revised) was 23/30, suggesting mild cognitive impairment (normal range is above 21/30) [5]. Routine laboratory data were all within normal limits including those for syphilis, anti-human T cell leukemia virus 1 antibody, thyroid function, vitamin $B_{12}$ and folic acid. The cerebrospinal fluid examination was unremarkable. Needle electromyography performed on the tongue, diaphragm, and anterior tibial muscle revealed mild chronic denervation. Magnetic resonance images (MRI) of the head revealed atrophy of the anterior part of the temporal and frontal lobes, slightly predominant on the right. Single photon emission computed tomography (SPECT) images disclosed decreased uptake of tracer, bilaterally, in the frontal and temporal lobes. After discharge she was observed as an outpatient, but her mental condition and motor symptoms gradually deteriorated. About five months after discharge, gastrostomy was performed for severe dysphagia. At which time, she had tetraparesis and could not respond to the examiner's instructions. She was transferred to the nursing hospital. Tracheostomy was not performed, and artificial ventilation not administered at the request of her family. The total duration of her illness was 18 months and she died of sudden respiratory failure.

## 3. Analysis of language function

Japanese language uses two distinct writing systems: kana characters, composed of simple phonograms with unambiguous phonetic readings, and kanji characters, a system of several thousand morphograms or ideograms.

On admission, her speech showed severe dysarthria and little intonation, and sounds and syllables were inconsistent. She occasionally had telegraphic speech and paraphrasia during spontaneous speech. The results of the WAB, examined during the first two weeks of admission, revealed spontaneous speech was non-fluent due to severe dysarthria. Repetition was mildly impaired. Although she could repeat nine words (each word was composed of two to ten characters) and two short sentences (each sentence was composed of eight and nine characters) without an error, she made two errors in repeating a long sentence composed of 20 characters, i.e. "新しい甘酒を五本のひょうたんに入れなさい" (Put fresh sweat alcohol into five gourds, in English). Object naming was mildly impaired (18/20), however, she could name all objects given phonemic cues. Sentence completion and responses in conversation were slightly impaired (8/10 and 8/10, respectively). As shown in Yes/No questions (60/60) and auditory word recognition (59/60), comprehension was not impaired.

In contrast to the results described above, her writhing was severely impaired. Although writing speed was slow, her formation of written characters showed no distortion. She made five errors in writing the same sentence as used in repetition: "新しい甘酒を五本のひょうたんに入れなさい" (she made two errors in repetition). She made four errors during writing six kanji words (each word was composed of two kanji characters), two errors in six kana words (each word was composed of three or four kana characters), and thirteen errors in four sentences (each sentence was composed of nine or ten characters). Most of the errors were omission of kana characters and adjuncts, such as postpositional particles. For example: "山上１本立て居す" instead of "山の上に木が１本立って居ます". The sentence is written, as normal, using a mixture of kanji and kana, and an English translation is: "There is a tree on the mountain". "の" and "に" are both particles, written in kana letters. And "木が" is the subject of the sentence, composed of "木"("tree" ) and "が"( a postpositional particle). "ま" is one of the so-called "okurigana", or kana characters added after kanji to indicate inflection. Other types of errors were substitution or addition of letters. Her ability to copy both letters and sentences was unimpaired. She could copy the sentence "新しい甘酒を五本のひょうたんに入れなさい" without an error.

After about three months, during follow up at the outpatient clinic, perseverative errors and paragraphia emerged. Moreover, morphology of her written characters became slightly distorted.

When tube feeding was necessary, she was no longer able to hold writing implements.

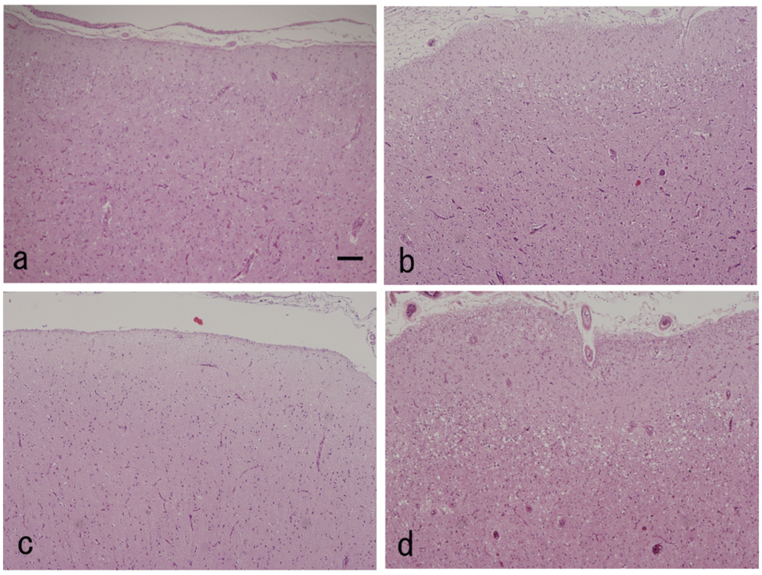

Fig. 1. Superficial spongiosis of the left frontal lobe cortices. a: superior frontal gyrus, b: middle frontal gyrus, c: inferior frontal gyrus, d: precentral gyrus. Severe spongiosis in the middle frontal (b) and precentral (d) gyri are shown. HE stain. Scale bar = 400 $\mu$m.

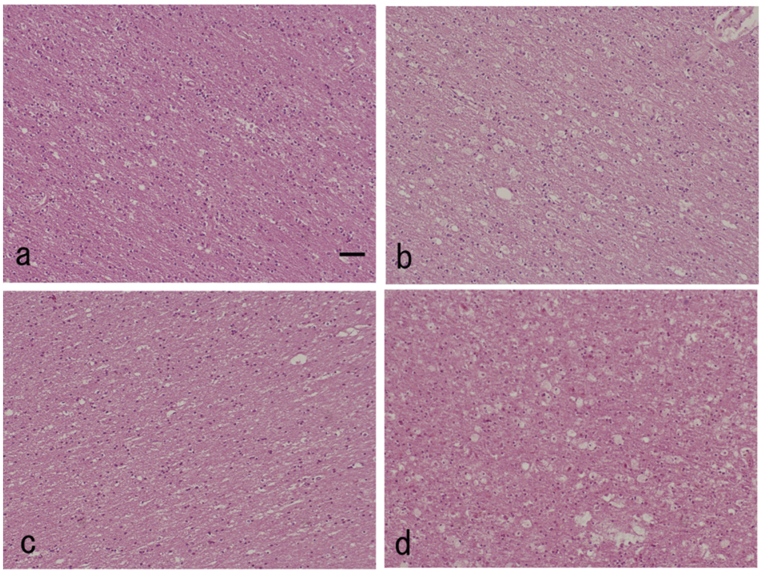

Fig. 2. Subcortical white matter of the left frontal lobe. a: superior frontal gyrus, b: middle frontal gyrus, c: inferior frontal gyrus, d: precentral gyrus. Numerous macrophages are observed in the middle frontal (b) and precentral (d) gyri. HE stain. Scale bar = 200 $\mu$m.

## 4. Neuropathological findings

General pathological examination revealed severe thinning of the diaphragma.

The brain weighed 1060 g prior to fixation. Macroscopic examination revealed circumscribed atrophy of the anterior temporal and frontal lobes bilaterally. Both lateral ventricles were slightly dilated. The substantia nigra was significantly depigmented.

Sections of paraffin-embedded tissue were stained with hematoxylin and eosin (H & E), Klüver-Barrera, and Bodian stains. In addition, immunohistochemistry for selected areas was performed using anti-ubiquitin (Dako; Japan; 1:100), anti-tau (AT8; Innunogenetics; 1:1000), and anti-phosphorylated-TDP-43 (p403/404,

Table 1
Summary of lesion distribution

| lesion (gyrus) | neuronal loss | superficial spongiosis | macrophage invasion | TDP pathology |
|---|---|---|---|---|
| superior frontal | mild | moderate | none | mild |
| middle frontal | mild | severe | severe | moderate |
| inferior frontal | mild | mild | none | mild |
| precentral | severe | severe | severe | moderate |

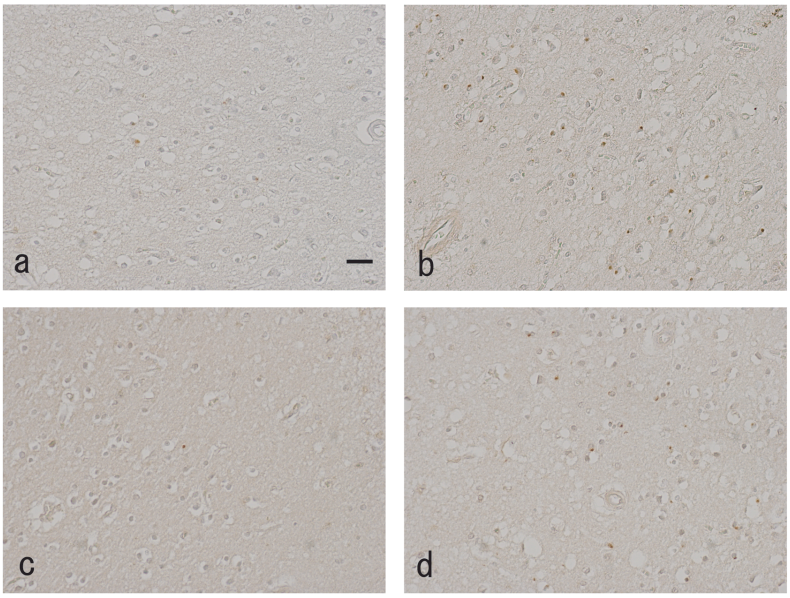

Fig. 3. Neuronal intra-cytoplasmic inclusions in the cortical neurons. a: superior frontal gyrus, b: middle frontal gyrus, c: inferior frontal gyrus, d: precentral gyrus. Numerous inclusions can be seen in the middle frontal (b) and precentral (d) gyri. Anti-phosphorylated-TDP-43-immunostain. Scale bar = 80 $\mu$m.

Cosmo Bio; California; 1:1000) antibodies.

Superficial spongiosis, neuronal loss, gliosis and rarefaction of the neuropil were observed in the frontal and temporal lobe cortices, especially in the precentral gyrus and posterior part of the middle frontal gyrus bilaterally (Fig. 1). Superficial spongiosis was also observed in the other frontal cortex, but to a lesser degree. Plenty of macrophages were observed in the subcortical white matter of these areas (Fig. 2). There were several clusters of macrophages in the deeper cortical layer of the precentral gyrus, indicating loss of Betz cells. Moderate neuronal loss was observed in the transitional area between CA1 and subiculum. Only a few neurofibrillary tangles were observed in the parahippocampal gyrus. No pathological change was observed in the left parietal and occipital lobes.

The pyramidal tracts showed almost complete axonal loss in the bilateral cerebral peduncles and pyramids. Neurons in the hypoglossal and ambiguous nuclei were preserved in number, however a few Bunina bodies were observed in the remaining neurons. The substantia nigra showed severe neuronal loss and gliosis with no Lewy bodies. The locus ceruleus showed mild neuronal loss and two Lewy bodies. The cerebellum was unremarkable. There were no neurofibrillary tangles in the brainstem or cerebellum.

In the spinal cord, moderate to severe loss of anterior horn cells and several Bunina bodies in the remaining neurons were observed. The pyramidal tracts showed almost complete axonal loss in the lateral columns. The posterior columns were unremarkable. Neurons in Onuf's nucleus were preserved in number.

Immuno-histochemistry showed ubiqutin and TDP-43-positive and tau-negative neuronal intracytoplasmic inclusions in the hipocampal dentate gyrus and frontal lobe cortex (Fig. 3). We detected these inclusions in the middle frontal and precentral gyri much more than in other frontal lobe cortices. Only a small number of TDP-43 positive dystrophic neurites were observed.

TDP-43 positive neuronal intranuclear inclusions and glial cells were not observed.

Summary of the pathological changes including immunohistochemistry are shown in Table 1.

## 5. Discussion

The presented case features a combination of progressive pseudobulbar palsy, frontal lobe type dementia, pyramidal tract signs and progressive amyotrophy. MRI revealed atrophy of the frontal and temporal lobes. Decreased blood flow in the frontal and temporal lobes was demonstrated by SPECT images. Pathological features of the case are: degeneration of both upper and lower motor neurons, degeneration of frontal and temporal lobes, Bunina bodies of the remaining lower motor neurons and TDP-43 pathology. These features are fully compatible with the diagnosis of ALS-D.

In progressive nonfluent aphasia, agraphia with effortful writing containing spelling error and agrammatism may be observed [6]. Agrammatism refers to omission or incorrect use of grammatical terms, including articles, prepositions, auxiliary verbs, inflexions, and derivations. From an initial stage, the presented patient showed progressive speech output disorder due to pseudobulbar palsy. Although telegraphic speech and occasional paraphasia were observed, frequency of such errors was quite less than that of writing errors. As shown in the WAB result, comprehension, naming and repetition were almost preserved when obvious agraphia was observed. Therefore, we believe the agraphia observed in our case did not derive from aphasia. Although perseverative errors were observed at a late stage, this can be attributed to frontal lobe dysfunction.

Omission of kana letters in Japanese and related syntactic errors in English have been reported in ALS with or without dementia [2,3,7,8]. The writing errors observed in our case concur well with previous descriptions. Although the lesion associated with pure agraphia is thought to be in the left frontal or parietal lobe [9], detailed clinicopathological analysis of ALS is unreported. A single autopsied case from the United States suggests that the left frontal or parietal lobe may be linked with the lesion responsible for syntactic errors in writing [7]. However, pathological findings related to agraphia were not described.

Our case reveals a left frontal lobe lesion with emphasis on the caudal part of the middle frontal gyrus, combined with immuno-histochemistry and other ALS-related indicators. Frontal and temporal lobe lesions including the hippocampal dentate gyrus have already been associated with dementia symptom in ALS-D [1]. A detailed description of each neuropsychological symptom and lesion remains to be elucidated, however.

The posterior part of the left middle frontal gyrus (Exner's area) is thought to play a pivotal role in writing letters and lesions there to cause agraphia [10,11]. Only a small number of cases of pure agraphia due to circumscribed lesions in Exner's area have been reported thus far [12–15]. Reports from Japan reveal phonological errors such as omission and paragraphia of kana letters associated with Exner's area lesion in cerebrovascular disease [15–17]. These errors concur well with the writing disorders observed in our case.

In the case described here, lesion distribution in the left frontal lobe was concentrated in Exner's area and no pathological change was observed in the left parietal lobe, which is another lesion associated with agraphia. We believe this is the first report that links clinicopathology in progressive agraphia without aphasia and detailed pathological examination including immunohistochemistry. Of course, further accumulation of clinicopathological examination focusing ALS-D and agraphia would be required to clarify our hypothesis.

## Acknowledgements

This study was supported by a grant from the Tamagawa University Center of Excellence from the Ministry of Education, Culture, Sports, Science and Technology (MEXT) and the Core Research for Evolutionary Science and Technology (CREST; No. 17022035) and by a Grant-in-Aid for Scientific Research on Priority Areas–System Study on Higher-order Brain Functions from MEXT (No. 20020026). This study was also supported in part by a Showa University Grant-in-Aid for Innovative Collaborative Research Projects and a Special Research Grant-in-Aid for Development of Characteristic Education from MEXT.
